# Iron‐Sensitive MRI Reveals Individual Differences in Pain Modulation Rather Than Categorical Markers in Chronic Pain and Migraine

**DOI:** 10.1155/prm/5063039

**Published:** 2026-10-12

**Authors:** Omar Ydlibi, Mona Salehi Ravesh, Florian Bernerth, Burkhard Maedler, Olav Jansen, Janne Gierthmühlen, Sönke Peters

**Affiliations:** ^1^ Department of Radiology and Neuroradiology University Hospital of Schleswig-Holstein Kiel Schleswig-Holstein Germany uksh.de; ^2^ Department of Medical Physics Faculty of Physics Technical University of Dortmund Dortmund North Rhine-Westphalia Germany; ^3^ Department of Pain Research and Therapy University Hospital of Schleswig Holstein Kiel Schleswig-Holstein Germany uksh.de; ^4^ Clinical Science Philips Healthcare Hamburg Germany philips.com; ^5^ Interdisciplinary Pain Unit Department of Anaesthesiology and Surgical Intensive Care Medicine University Hospital of Schleswig Holstein Kiel Schleswig-Holstein Germany uksh.de

**Keywords:** conditioned pain modulation, globus pallidum, periaqueductal grey, pupil reflex dilation, quantitative magnetic susceptibility mapping, quantitative sensory testing, *R*_2_^∗^ mapping, red nucleus

## Abstract

**Objective:**

To determine whether regional iron levels are associated with endogenous pain‐inhibitory function and whether iron‐sensitive MRI techniques, quantitative magnetic susceptibility (QSM) and ${R_{2}}^{*}$ mapping capture differences in midbrain iron content in the globus pallidus (GP), periaqueductal grey (PAG), and red nucleus (RN) across chronic pain (CP), migraineurs (M), and healthy volunteers (HV).

**Methods:**

Sixty participants (20 CP, 20 M, and 20 HV) underwent a 3T MRI examination. Pain modulatory and autonomic function was assessed using conditioned pain modulation (CPM) and pupil reflex dilation (PRD) across all participants (*n* = 60), whereas quantitative sensory testing (QST) and pain sensitivity questionnaire (PSQ) were assessed in CP and M only (*n* = 40). Group differences and iron–pain relationships were evaluated using ANOVA or Kruskal–Wallis tests and linear regression models.

**Results:**

Distinct region‐ and modality‐specific relationships were observed between iron‐sensitive MRI measures and pain‐related outcomes. ${R_{2}}^{*}$ RN was associated with absolute and relative CPM effects. QSM PAG was associated with temporal summation and showed a trend‐level association with subjective pain sensitivity (PSQ‐minor). Autonomic reactivity PRD was associated with ${R_{2}}^{*}$ measures in both regions. ${R_{2}}^{*}$ RN was related to PRD during heat stimulation, whereas ${R_{2}}^{*}$ PAG was related to PRD during heat and cold‐water conditioning. QSM/${R_{2}}^{*}$ RN were further associated with mechanical pain threshold (MPT) and mechanical pain sensitivity (MPS). Group‐level differences were, however, minimal between CP, migraine, and HV cohorts.

**Conclusion:**

Modality‐specific associations between iron‐sensitive MRI measures and descending inhibition, nociceptive gain, and autonomic reactivity were observed. These findings indicate that QSM and ${R_{2}}^{*}$ mapping together capture individual variation in pain‐modulatory physiology, supporting the concept of an iron‐related biotype of pain modulation rather than a categorical marker. In contrast, regional iron content did not differentiate CP, M, and HV groups.

## 1. Introduction

Chronic pain (CP) and migraine (M) are characterised by altered sensory processing, impaired descending pain control, and dysregulated autonomic responses [1, 2]. Yet, the neurobiological mechanisms that link these functional changes to underlying brain structure remain only partly understood. Iron homeostasis is central to neuronal metabolism, oxidative balance, and neurotransmission [3], and disruptions in iron regulation within deep brain nuclei and the brainstem have been associated with neurodegeneration, motor dysfunction, and altered nociceptive signalling. Within this context, the periaqueductal grey (PAG) and red nucleus (RN) are of particular interest. Both structures lie at the core of midbrain circuits involved in sensory integration and descending pain modulation [4, 5]. The globus pallidus (GP), though primarily involved in motor regulation and basal ganglia function, was included as a high‐iron comparison region. The PAG exerts inhibitory control over spinal nociceptive pathways and coordinates autonomic responses [4, 6], whereas the RN integrates cerebellar and cortical inputs, contributing to sensorimotor function and potentially to nociceptive processing [5]. Previous magnetic resonance imaging (MRI) work using T_2_‐weighted imaging (T_2_w) has reported signal changes in the PAG and RN in chronic migraine, findings that have been interpreted as consistent with increased iron deposition [7]. Such signal changes have been suggested as reflecting cumulative metabolic stress, leading to altered activity within pain‐modulatory circuits. However, results across studies remain heterogeneous, and it remains unclear whether similar alterations extend beyond migraine to other CP conditions. Moreover, the functional relevance of regional iron content for pain sensitivity or pain‐modulatory capacity has received limited attention. To our best knowledge, no study has directly compared patients with CP and M to healthy volunteers (HV) in terms of midbrain iron content and its relationship to behavioural and physiological markers of pain regulation. This represents an important gap, given that CP and migraine share several pathophysiological features but may also diverge in their neurobiological substrates. To address this, we combined quantitative magnetic susceptibility (QSM) and ${R_{2}}^{*}$ MRI to quantify regional brain iron in the PAG, RN, and GP across all three groups. We further examined how iron levels in these regions relate to multiple indices of pain processing, including quantitative sensory testing (QST) [8], conditioned pain modulation (CPM) [9], and autonomic reactivity measured via pupil reflex dilation (PRD) [10]. Self‐reported pain sensitivity, assessed using a pain sensitivity questionnaire (PSQ) [11], was included as a complementary psychometric measure. The primary aim of this study was to examine whether regional midbrain iron content is associated with individual differences in pain‐modulatory, sensory, and stimulus‐evoked autonomic function across participants. As a secondary and exploratory aim, we examined whether iron‐sensitive MRI measures differ between CP patients, M, and HV. We hypothesised that higher iron content in pain‐modulatory midbrain regions, particularly in the PAG and RN, would be associated with reduced descending pain inhibition, enhanced nociceptive gain, and stronger autonomic responses to noxious stimulation.

## 2. Methods

### 2.1. Participants

Participants were prospectively recruited from the Departments of Neurology (Pain Research) and Radiology and Neuroradiology at the University Hospital Schleswig‐Holstein, Campus Kiel, as well as from the surrounding community of Authors. All individuals were over 18 years old, medically screened, received written study information, and provided written informed consent. Migraine patients met the International Classification of Headache Disorders (ICHD‐3) criteria for episodic or chronic migraine. CP patients had chronic non‐neuropathic pain outside the head without evidence of peripheral or central nervous system disease, as confirmed by a neurologist, ensuring that no included participant had conditions that could affect sensory, autonomic, or MRI‐based measurements. HV reported no history of neurological or CP disorders. Exclusion criteria comprised pregnancy or breastfeeding, cancer history, implanted electronic devices (e.g., pacemaker, defibrillator, pain pump), rheumatoid arthritis, and any neurological disorder (e.g., stroke, Parkinson’s disease, multiple sclerosis, neuromuscular disease, peripheral neuropathy, or other structural central nervous system (CNS) pathology). Individuals with muscle or nerve disorders affecting sensory testing or autonomic responses, as well as those with current or past psychiatric disorders, were excluded. Migraine patients with additional acute or CP conditions were not eligible. Likewise, CP participants did not fulfil diagnostic criteria for migraine. Within the CP cohort, nine participants reported single‐site pain, and eleven reported multi‐site pain. Because pain localisation varied across participants, pain distribution was characterised according to the number of affected sites rather than laterality. The study originally aimed to recruit 40 M patients, 40 CP patients, and 20 HV. Due to screening exclusions and coronavirus disease (COVID)‐19 restrictions, the final sample comprised 20 participants in each group. Data were pseudonymised (PD001–PD062) and stored according to institutional and general data protection regulation (GDPR) requirements. Two participants (PD006 and PD007) were excluded due to incomplete outcome data. Lifestyle and clinical variables (age, gender, height, weight, smoking status, alcohol use, and medication) were documented to provide clinical context. Additional migraine‐specific information (attack frequency, intensity, aura, prophylaxis, symptoms) was also recorded. However, because these variables applied only to migraine participants, and the sample consisted almost entirely of episodic migraine (19 EM, 1 CM), they were not included in the primary analyses, which focused on dimensional associations between QSM/${R_{2}}^{*}$ and pain‐modulatory outcomes and group comparisons of iron content.

### 2.2. MRI Examination

An MRI was performed on a whole‐body MRI system (3T, Ingenia CX, Philips Healthcare, Best, Netherlands) using a 32‐channel digital (d) Stream head coil. Table 1 shows our MRI protocol, which included 3D multi‐fast field echo (mFFE) pulse sequences to quantify magnetic susceptibility based on QSM reconstruction and clinical standard pulse sequences to assess anatomical structure. All datasets were reviewed by a senior neuroradiologist (Sönke Peters) to exclude clinically relevant abnormalities.

**TABLE 1:** MRI protocol parameters: 3D‐mFFE and clinical pulse sequences of the study.

Protocol parameters	3D FLAIR (VISTA)	3D T_1_w TFE	2D T_2_w (DRIVE)	3D mFFE
TE (ms)	338	3.3	80	3.32–27.54 (7 TEs with an increment of 4.04 ms)
TR (ms)	8000	7.6	5986	31
TI (ms)	2400	—	—	—
Slice orientation	Sagittal	Sagittal	Axial	
Field of view (mm^2^ or mm^3^)	250 × 250 × 250	240 × 240 × 170	220 × 174 × 147	190 × 133 × 135
Acquired pixel size (mm^2^, mm^3^)	1.1 × 1.1 × 1.2	1.0 × 1.0 × 1.0	0.50 × 0.68 × 2.0	0.7 × 0.7 × 1.2
Slice thickness (mm)	2.0	2.0	2.0	1.2
Reconstructed pixel/voxel size (mm^2^ or mm^3^)	0.33 × 0.33 × 0.6	0.47 × 0.47 × 1.0	0.2 × 0.2 × 2.0	0.6 × 0.6 × 0.6
SENSE factor P reduction AP)/S reduction (RL)	—/—	—/—	—/—	2.0/1.20
WFS (pix)/BW (Hz)	0.895/485.2	0.525/826.7	2.1/206.9	0.70/621.0

Abbreviations: AP, Anterior‐Posterior; BW, Bandwidth; DRIVE, Driven Equilibrium; FLAIR, Fluid Attenuated Inversion Recovery; FOV, Field of View; mFFE, multi‐fast field Echo; RL, Right‐Left; T_1_w, T_1_‐weighted imaging; T_2_w, T_2_‐weighted imaging; TE, Time of Echo; TFE, Turbo Field Echo; TI, Time of Inversion; TR, Time of Repetition; VISTA, Volume Isotropic Turbo Spin‐Echo Acquisiton; WFS, Water‐Fat‐Shift.

### 2.3. Quantitative Analysis of MRI Datasets

QSM and ${R_{2}}^{*}$ (= 1/${T_{2}}^{*}$) maps were calculated from 3D mFFE data [12]. For QSM, phase data were echo‐wise unwrapped with Laplacian‐based phase unwrapping [13, 14]. Then background field removal was performed for each echo using the variable‐kernel sophisticated harmonic artefact reduction for phase data (V‐SHARP) [13, 14] method with a kernel size of up to 12 mm. For this background field removal step, for each echo, a brain mask was generated with the FSL Brain Extraction Tool [15] using the magnitude data of the corresponding echo. Finally, echoes were averaged [16] using only nonzero pixels from each echo, and susceptibility maps were calculated from local phase data using the streaking artefact reduction for QSM (STAR‐QSM) algorithm [17]. ${R_{2}}^{*}$ maps were calculated with the algorithm for fast monoexponential fitting based on autoregression on linear operations (ARLO) from mFFE magnitude data of all seven echoes [18]. Regional quantification was conducted using the Medical Imaging Interaction Toolkit program (MITK, DKFZ, Heidelberg, Germany), which provides stable voxel statistics across repeated measurements and was therefore used for regions‐of‐interest (ROI)‐based analyses. ROIs were manually drawn bilaterally in the GP and RN as well as the PAG, guided by anatomical landmarks and validated with input from our experienced senior neuroradiologist. ROI delineation and quantification were performed in each participant’s native image space rather than in a standardised template space. For statistical analyses, the median QSM (ppm) and ${R_{2}}^{*}$ (1/s) values within each ROI were used. Left and right GP and RN ROIs were represented by a single bilateral regional value for each participant. For each ROI, MITK extracted descriptive statistics (mean, median, variance, standard deviation, minimum, maximum, range, skewness, and kurtosis) of the voxel values. These parameters characterised regional magnetic susceptibility and ${R_{2}}^{*}$ relaxation characteristics, serving as markers of local iron content (Figure 1).

**FIGURE 1:**
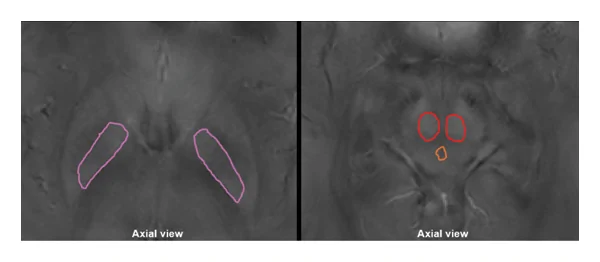
Example showing the manual placement of bilateral ROIs in the GP and RN as well as in PAG in a QSM sequence. Quantitative susceptibility mapping is abbreviated to QSM, globus pallidus to GP, periaqueductal grey to PAG, red nucleus to RN, and region of interest to ROI.

### 2.4. CPM

CPM was assessed to evaluate endogenous descending pain inhibition using a trigeminal‐focused [19, 20] protocol. A 30 × 30 mm thermode (Medoc Advanced Medical Systems, Israel) was placed on the right forehead to deliver an individually calibrated heat test stimulus (TS). The stimulus lasted 30 s, and pain intensity was rated at 10, 20, and 30 s on a 0–100 numerical rating scale (NRS). After a brief pause, the conditioning stimulus (CS) was applied by placing a cool pack (mean temperature −8.1 ± 2.9°C) on the left jaw/mandible. This created a ‘pain inhibits pain’ [9, 21] setup in which the cooling acted as the CS while heat on the contralateral forehead served as the TS. Due to a slight timing offset, participants first rated the cooling sensation at 30 s, followed by TS ratings at 40 and 50 s; an additional TS and CS rating was obtained at 60 s separately. After removal of the CS, the heated TS was applied again for 30s to assess post‐conditioning effects. CPM effects were calculated from pain intensity ratings only. The absolute CPM effect was calculated as the change in pain ratings in NRS units (baseline TS‐conditioned TS). The relative CPM effect quantified the proportional change in pain relative to baseline, expressed as a unitless fractional value to account for individual differences in initial pain sensitivity. Together, these indices capture both the magnitude (absolute) and efficiency (relative) of descending pain modulation across all participants (*n* = 60) [9, 21].

### 2.5. QST

QST was performed according to the standardised protocol of the German Research Network on Neuropathic Pain (Deutscher Forschungsverbund Neuropathischer Schmerz e.V., DFNS) [22] to assess thermal and mechanical sensitivity in M and CP patients (*n* = 40). Thermal and mechanical QST parameters were analysed using EQUISTA, the DFNS‐standard software that automatically extracts thresholds and computes sensitivity metrics from recorded stimulus‐response data. All QST parameters were analysed in their log‐transformed values, as provided by the software. Thermal thresholds were obtained using a thermosensory stimulator (Medoc Advanced Medical Systems, Israel) with the cold pain threshold (CPT) defined as the temperature at which a decreasing stimulus first became painful, and heat pain threshold (HPT) as the temperature at which increasing heat first elicited pain. Mechanical testing using DFNS pinpricks, von Frey filaments, and soft brushes assessed mechanical pain threshold (MPT), mechanical pain sensitivity (MPS), and dynamic mechanical allodynia (DMA). Temporal pain summation was quantified using the wind‐up ratio (WUR), calculated by comparing NRS pain ratings of a single pinprick stimulus with those of a series of repeated stimuli [8].

### 2.6. PRD

PRD was recorded as an objective autonomic marker of nociceptive processing. PRD assessment was performed across all participant groups, including CP, M and HV (*n* = 60). Pupillary responses were measured continuously using a hand‐held pupillometer (IDMED, Marseille, France) during three conditions: bright light exposure, heat stimulation, and combined heat stimulation with cold‐water immersion. Measurements were taken from the eye with better self‐reported visual acuity. PRD was quantified as relative change of pupil diameter [10] to baseline, with greater change indicating stronger sympathetic activation. This was then shown as a percentage. PRD assessment was carried out during the standard forearm‐based CPM protocol. A light stimulus was first used to establish a baseline pupillary reflex. The second PRD measurement was recorded during thermal stimulation of the right forearm and the third during conditioning, when cold‐water immersion of the left hand was paired with the same heat stimulus. This design enabled examination of whether autonomic responses to pain are modulated under a conditioning stimulus. Consistent with recent findings, PRD typically increases during noxious stimulation and attenuates under CPM‐like conditions [10], supporting its role as a complementary physiological index of pain‐related autonomic reactivity and potential modulation.

### 2.7. PSQ

Subjective pain sensitivity was assessed using PSQ [11], a self‐report measure that quantifies train‐like sensitivity to imagined everyday situations. The questionnaire was administered to CP and M participants only (*n* = 40). Participants rated the perceived painfulness of each situation on a numerical scale ranging from 0 (no subjective pain/not painful at all) to 10 (worst subjective pain imaginable). Higher scores indicate greater subjective pain responsiveness. Subscale scores were calculated for PSQ‐Minor, PSQ‐Moderate, and PSQ‐Total. The PSQ‐Minor subscale reflects sensitivity to mild, everyday discomfort, PSQ‐Moderate captures typical daily pain experiences, and PSQ‐Total represents overall pain sensitivity across items of varying intensity. All PSQ subscales were included in subsequent analyses.

### 2.8. Statistical Analysis

All statistical analyses were performed using Jamovi (Version 2.3.28, Sydney, Australia). Descriptive statistics were computed for all main variables (mean, median, standard deviation, minimum and maximum values, skewness, and kurtosis). Data distribution and potential outliers were examined visually using boxplots. Normality was assessed with the Shapiro–Wilk test, and variables were summarised as mean ± SD for normally distributed data or median [IQR] for non‐normal data. To evaluate whether regional brain iron levels (QSM/${R_{2}}^{*}$) were associated with pain‐related outcomes (CPM, PRD, QST, PSQ), linear regression models were built separately for the QSM and ${R_{2}}^{*}$ datasets. Each pain measure served as the dependent variable. Regional iron values from GP, PAG, and RN were included as independent variables. Clinical groups (CP, M and HV) remained as categorical independent factors. Omnibus ANOVA *p* values were used to determine whether regional iron showed significant associations with each pain measure. Model coefficients were further examined to identify which regional iron measures and group effects contributed to each model. Collinearity diagnostics indicated no evidence of multicollinearity (Variance Inflation Factor, VIF ≈ 1). To assess the potential influence of demographic factors, all dimensional regression analyses were repeated, including age and sex as covariates. The clinical group was entered as a categorical main effect in all models. Analyses involving CPM and PRD outcomes included the full cohort (*n* = 60), whereas analyses involving QST and PSQ outcomes were restricted to the CP and M groups (*n* = 40). The results of these dimensional analyses are presented in Table 2. *p* values were interpreted descriptively, and no formal correction for multiple comparisons was applied.

**TABLE 2:** Linear regression analyses revealing associations between regional brain iron (QSM and ${\text{R}}_{2}^{*}$) and behavioural pain measures (CPM, QST, PRD, and PSQ).

Outcome	QSM‐GP (*p* value)	${R_{2}}^{*}$‐GP (*p* value)	QSM‐PAG (*p* value)	${R_{2}}^{*}$‐PAG (*p* value)	QSM‐RN (*p* value)	${R_{2}}^{*}$‐RN (*p* value)	*p* values for QSM			*p* values for ${R_{2}}^{*}$		
							CP vs. HV	M vs. HV	CP vs. M	CP vs. HV	M vs. HV	CP vs. M
*Conditioned pain modulation (CPM)*												
Absolute (ΔNRS)	0.985	0.472	0.334	0.150	0.213	0.015**^∗^**	0.078^†^	0.971	0.072^†^	0.037**^∗^**	0.949	0.038**^∗^**
Relative fractional change	0.708	0.705	0.397	0.170	0.301	0.022**^∗^**	0.070^†^	0.835	0.043**^∗^**	0.035**^∗^**	0.809	0.017**^∗^**

*Pupil reflex dilation (PRD) under stimulation in %*												
BL	0.510	0.615	0.478	0.148	0.715	0.832	0.085^†^	0.455	0.336	0.109	0.448	0.364
Heat	0.597	0.761	0.876	0.970	0.754	0.016**^∗^**	0.892	0.946	0.836	0.770	0.877	0.884
Heat and cold water	0.674	0.761	0.999	0.023**^∗^**	0.834	0.092^†^	0.219	0.868	0.290	0.125	0.701	0.229

*Quantitative sensory testing (QST)*												
DMA	0.122	0.112	0.693	0.244	0.277	0.238	N.A.	N.A.	0.061^†^	N.A.	N.A.	0.140
CPT (°C)	0.260	0.053^†^	0.622	0.390	0.704	0.477	N.A.	N.A.	0.471	N.A.	N.A.	0.730
HPT (°C)	0.431	0.341	0.422	0.557	0.362	0.575	N.A.	N.A.	0.007**^∗^**	N.A.	N.A.	0.005**^∗^**
MPT (mN)	0.655	0.145	0.094^†^	0.326	0.020**^∗^**	0.298	N.A.	N.A.	0.237	N.A.	N.A.	0.161
MPS	0.812	0.546	0.148	0.072^†^	0.372	0.030**^∗^**	N.A.	N.A.	0.096^†^	N.A.	N.A.	0.051^†^
WUR	0.303	0.268	0.009**^∗^**	0.180	0.303	0.634	N.A.	N.A.	0.025**^∗^**	N.A.	N.A.	0.047**^∗^**

*Pain sensitivity questionnaire (PSQ scores)*												
Minor	0.093^†^	0.486	0.072^†^	0.451	0.143	0.812	N.A.	N.A.	0.122	N.A.	N.A.	0.364
Moderate	0.082^†^	0.262	0.355	0.189	0.688	0.635	N.A.	N.A.	0.367	N.A.	N.A.	0.869
Total	0.120	0.368	0.199	0.328	0.733	0.486	N.A.	N.A.	0.244	N.A.	N.A.	0.741

*Note:* Omnibus *p* values indicate whether each iron region was related to the respective outcome. Significant or trend‐level *p* values of diagnostic group comparisons (CP, M, and HV) from model coefficients are shown for QSM and ${R_{2}}^{*}$ separately. Quantitative susceptibility mapping is abbreviated to QSM, conditioned pain modulation to CPM, quantitative sensory testing to QST, pupil reflex dilation to PRD, pain sensitivity questionnaire to PSQ, chronic pain to CP, migraine to M, and healthy volunteer to HV, not applicable to N.A.

^∗^ *p* < 0.05.

^†^Trend (0.05–0.10).

As a secondary exploratory analysis, group differences were evaluated using Welch’s ANOVA for normally distributed variables and the Kruskal–Wallis test for non‐normally distributed variables. Post‐hoc comparisons were performed using Games–Howell tests for parametric data or DWASS–Steel–Critchlow–Fligner (DSCF) tests for non‐parametric data.

## 3. Results

Demographic and clinical data of the included participants of all groups (CP; M; HV) are provided in Table 3.

**TABLE 3:** Comparison of demographic and clinical characteristics across all groups.

**Characteristic**	**CP (*n* = 20)**	**M (*n* = 20)**		**HV (*n* = 20)**
		**19 (EM)**	**1 (CM)**	

Age, y/o	35.5 [27.8, 53.8]	34.0 [25.8, 48.3]	32.5 [27.0, 45.0]	
Gender (f/m)	13/7	14/6	11/9	
Pain localisation/type	Single‐site (9)/multi‐site (11)	EM (19)	CM (1)	—
Duration of chronic pain/Migraine (years)	3.5 ± 0.89	3.1 ± 1.65 (EM)	> 5 (CM)	—

*Note:* Age is presented as median [IQR]; duration of pain as mean ± SD; categorical variables (group and gender) are presented as counts (*n*). Chronic pain is abbreviated to CP, migraine to M, healthy volunteer to HV, episodic migraine to EM and chronic migraine to CM.

### 3.1. QSM and ${R_{2}}^{*}$

Group‐wise distributions of QSM and ${R_{2}}^{*}$ values in the GP, PAG, and RN, together with the corresponding group comparisons, are shown in Table 4 and Figures 2 and 3. Across regions, categorical group differences in QSM/${R_{2}}^{*}$ were limited. A significant group effect was observed only for QSM values in the GP (Kruskal–Wallis *p* = 0.04). Pairwise comparisons showed trend‐level differences between M vs. HV (*p* = 0.056), while CP vs. M and CP vs. HV did not reveal any significance. No significant group differences were detected for QSM values in the PAG or RN. For ${R_{2}}^{*}$ measures, no significant group differences were observed in GP, PAG, or RN. Overall, these findings suggest that QSM and ${R_{2}}^{*}$ measures showed limited ability to distinguish between clinical groups. GP was included as a high‐iron comparison region that is not primarily involved in descending pain modulation, allowing evaluation of the regional specificity of iron–pain associations.

**TABLE 4:** Group differences across QSM/${R_{2}}^{*}$ as well as all behavioural and sensory outcomes (CPM, QST, PRD, and PSQ) assessed using either ^1^Kruskal–Wallis test or ^2^Welch ANOVA (indicated by superscripts ^1^ and ^2^, respectively) depending on non‐normal/normal data distribution.

Domain	CP	M	HV	Group comparison *p* value ^1^ = KW, ^2^ = Welch ANOVA	Pairwise comparison		
					CP vs. HV	M vs. HV	CP vs. M
QSM of Midbrain [ppm]							
GP	0.087 [0.080, 0.10]	0.074 [0.062, 0.088]	0.096 [0.077, 0.106]	**0.040** ^**1**∗^	0.662	0.056^†^	0.141
PAG	−0.067 [−0.160, −0.05]	−0.127 [−0.209, −0.072]	−0.10 [−0.150, −0.074]	0.326^1^	0.536	0.352	0.492
RN	0.121 ± 0.101	0.151 ± 0.115	0.172 ± 0.08	0.222^2^	0.242	0.782	0.606

R_2_∗ of Midbrain [1/s]							
GP	38.8 [35.8, 41.5]	38.2 [36.3, 40.0]	38.4 [35.8, 40.6]	0.948^1^	0.986	0.994	0.934
PAG	13.7 [11.8, 16.4]	14.3 [12.8, 15.2]	13.6 [12.2, 15.3]	0.691^1^	0.890	0.696	0.878
RN	31 ± 3.61	32 ± 4.62	33.3 ± 4.65	0.247^2^	0.231	0.608	0.764

TS of forehead‐right side (°C)	47.4 ± 0.745	47.1 ± 1.12	47.6 ± 0.587				

Absolute CPM (ΔNRS)	4.5 ± 11.2	13 ± 15.4	11.3 ± 15.1	0.102^2^	0.281	0.926	0.144

Relative fractional change of CPM	0.061 ± 0.21	0.243 ± 0.28	0.190 ± 0.28	0.058^2†^	0.255	0.793	0.072^†^

*Forehead-right side*							
DMA	0	0	N.A.	0.144^1^	N.A.	N.A.	0.144
CPT (°C)	0.744 ± 1.16	0.547 ± 0.99	N.A.	0.566^2^	N.A.	N.A.	0.566
HPT (°C)	−0.79 ± 1.09	−0.34 ± 1.09	N.A.	**0.003** ^**2∗**^	N.A.	N.A.	**0.003** ^**∗**^
MPT (mN)	1.15 ± 1.43	0.695 ± 1.16	N.A.	0.281^2^	N.A.	N.A.	0.281
MPS	1.0 ± 1.79	0.141 ± 1.25	N.A.	0.086^2†^	N.A.	N.A.	0.086^†^
WUR	−0.479 ± 0.92	0.171 ± 1.11	N.A.	0.051^2†^	N.A.	N.A.	0.05^†^

*Pupil reflex dilation (PRD) under stimulation in (%)*							
BL	10.9 [7.73, 18.1]	8.6 [5.38, 16.9]	7.6 [3.45, 16.0]	0.210^1^	0.199	0.778	0.508
Heat	23.3 [9.97, 45.3]	15.3 [11.4, 31.3]	20.5 [15.1, 51.2]	0.467^1^	0.738	0.427	0.913
Heat and cold water	24.3 [10.3, 38.0]	10.3 [5.6, 31.1]	17.1 [11.6, 31.1]	0.526^1^	0.851	0.808	0.508

*Pain sensitive questionnaire (PSQ-cores)*							
Minor	2.43 [2.1–3.6]	2.64 [1.79–3.25]	N.A.	0.597^1^	N.A.	N.A.	0.597
Moderate	4.64 [3.6–5.93]	4.78 [3.96–5.11]	N.A.	0.797^1^	N.A.	N.A.	0.797
Total	3.79 [3.12–4.9]	4.11 [3.09–4.61]	N.A.	0.710^2^	N.A.	N.A.	0.710

*Note:* Variables are subsequently presented as median [IQR] or mean ± SD as appropriate. For each variable, the table reports the global omnibus *p* value and pairwise group comparisons (CP vs. HV, CP vs. M, M vs. HV). Significance markers indicate: ^∗^ *p* < 0.05; ^†^trend‐level (0.05 ≤ *p* < 0.10). Distributional characteristics are visualised in the corresponding boxplots (Figures 2, 3, 4, 5, 6, and 7). Quantitative susceptibility mapping is abbreviated to QSM, conditioned pain modulation to CPM, quantitative sensory testing to QST, pupil reflex dilation to PRD, pain sensitivity questionnaire to PSQ, chronic pain to CP, migraine to M, healthy volunteer to HV, not applicable to N.A.

**FIGURE 2:**
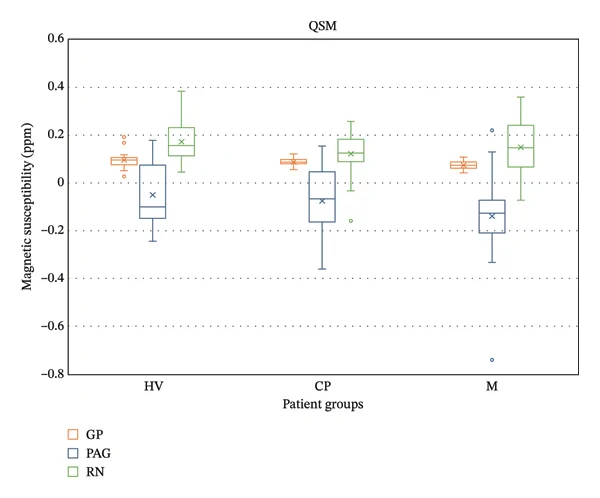
Distribution of measured iron content derived from QSM within the GP, PAG, and RN in all groups. Quantitative susceptibility mapping is abbreviated to QSM, globus pallidus to GP, periaqueductal grey to PAG, red nucleus to RN, chronic pain to CP, migraine to M, and healthy volunteers to HV.

**FIGURE 3:**
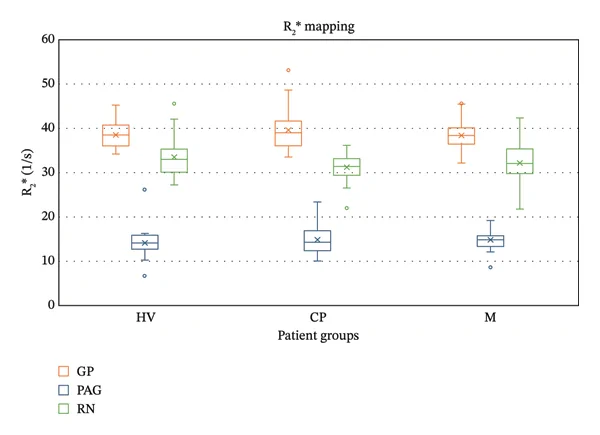
Distribution of measured iron content derived from $R_{2}^{*}$ maps within the GP, PAG, and RN in all groups. Globus pallidus is abbreviated to GP, periaqueductal grey to PAG, red nucleus to RN, chronic pain to CP, migraine to M, and healthy volunteers to HV.

### 3.2. CPM

Absolute and relative CPM effects are illustrated in Figure 4 and shown in Table 4. The absolute CPM effect was measured as the change in NRS and did not differ significantly between groups (*p* = 0.102). Relative CPM, shown as the fractional change accordingly, revealed a trend‐level group effect (*p* = 0.058), driven by a lower CPM effect in the CP group compared with HV and M. Across both CPM measures, M exhibited the largest inhibitory effects, HV showed intermediate values, and CP patients showed the smallest, or rather most attenuated, inhibitory responses. The absolute and relative CPM values therefore indicate the direction and magnitude of inhibitory pain modulation within each cohort. Positive values indicate pain inhibition, and negative values indicate pain facilitation.

**FIGURE 4:**
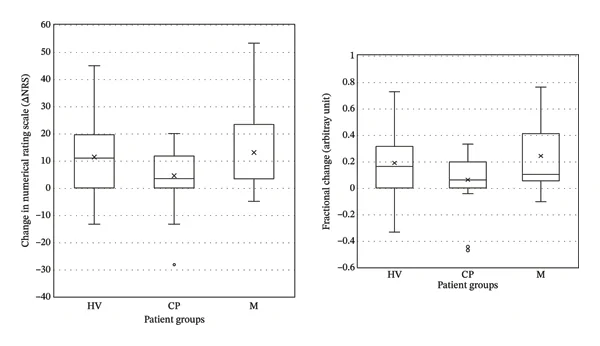
Distribution of measured absolute (left) and relative (right) CPM effects in the head region across all groups. Conditioned pain modulation is abbreviated to CPM, chronic pain to CP, migraine to M, and healthy volunteers to HV.

### 3.3. PRD

The distribution of the PRD responses (PRD after painful stimulus – base diameter of the pupil before stimulus) during bright light stimulation, heat stimulation, and combined heat with cold‐water conditioning is illustrated in Figure 5 and shown in Table 4. No significant group differences were observed for PRD under any condition.

**FIGURE 5:**
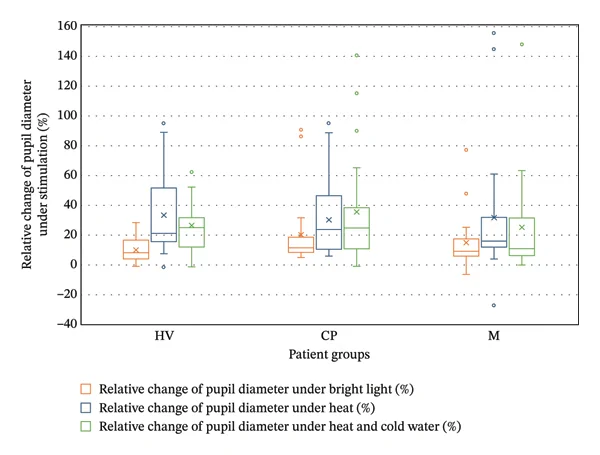
PRD responses during BL, heat stimulation, and the combined (H + CW) condition. The plots visualise differences in autonomic reactivity patterns between groups under increasing stimulus intensity. Pupil reflex dilation is abbreviated to PRD, bright light stimulation to BL, heat and cold water to H + CW, chronic pain to CP, migraine to M, and healthy volunteers to HV.

### 3.4. QST

All QST results from DFNS/EQUISTA are provided as log‐transformed scales (directly from raw threshold data) and are shown in Figure 6a–b and in Table 4. No group differences were observed for CPT (*p* = 0.566), MPT (*p* = 0.281), or DMA (*p* = 0.144). In contrast, HPT differed significantly between CP and M (Welch ANOVA *p* = 0.003), with CP patients showing lower thresholds. MPS (*p* = 0.086) and WUR (*p* = 0.051) showed trend‐level group effects, with CP patients tending towards greater mechanical sensitivity and enhanced temporal summation compared with migraine patients. These findings indicate altered nociceptive processing in CP, particularly for thermal and mechanical gain‐related measures.

**FIGURE 6:**
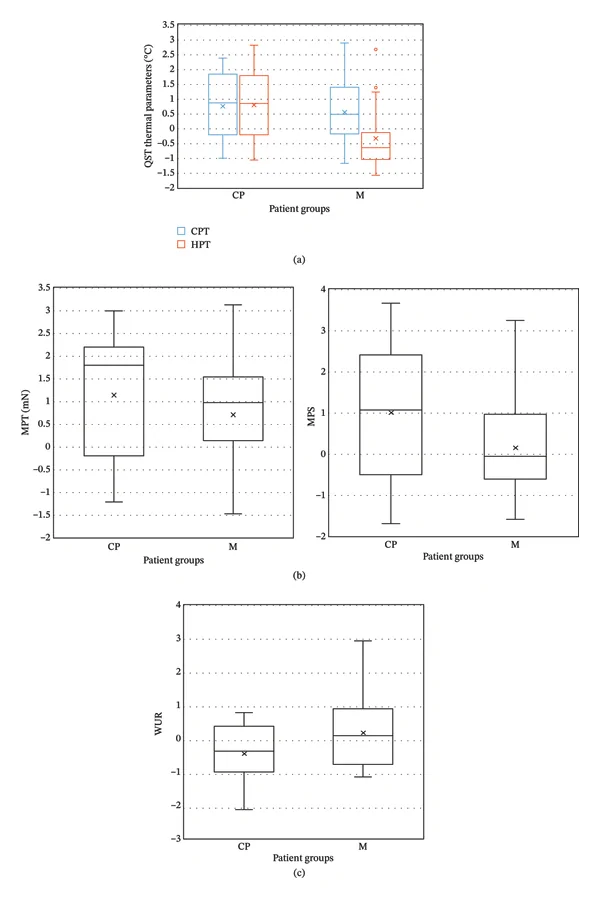
QST outcomes including (a) CPT and HPT, (b) MPT and MPS, and (c) WUR. Thermal and mechanical parameters are displayed on log‐transformed scales as provided by the DFNS/EQUISTA software. Plots above provide an overview of sensory and nociceptive sensitivity profiles across chronic pain and migraine patients. No boxplot is shown for DMA because all values were zero. Quantitative sensory testing is abbreviated to QST, dynamic mechanical allodynia to DMA, cold pain threshold to CPT, heat pain threshold to HPT, mechanical pain threshold to MPT, mechanical pain sensitivity to MPS, wind‐up ratio to WUR, chronic pain to CP, and migraine to M.

### 3.5. PSQ

Distribution of PSQ scores (minor = low‐intensity, mildly painful situations/moderate = more clearly painful but still given daily situations/total = mean of all painful items) is illustrated below in Figure 7 and shown in Table 4. No significant group differences were detected for all scores between CP and M participants (Table 4), indicating comparable levels of subjective pain sensitivity across all groups.

**FIGURE 7:**
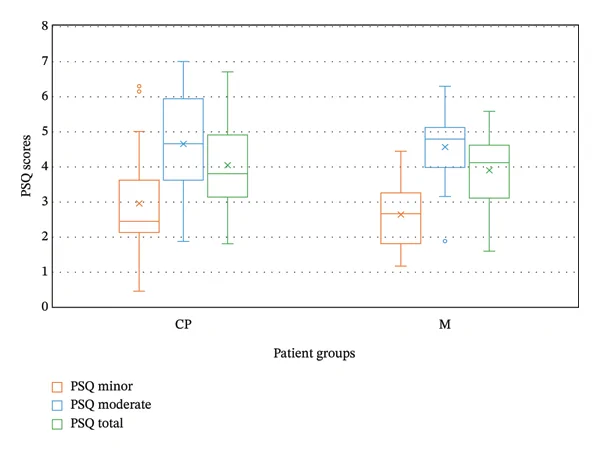
PSQ scores demonstrating subjective pain sensitivity across patient groups. Pain sensitivity questionnaire is abbreviated to PSQ, chronic pain to CP, and migraine to M.

### 3.6. Associations Between QSM/$R_{2}^{*}$ and Pain‐Related Outcomes

To address the primary study objective, we examined whether regional QSM and ${R_{2}}^{*}$ measures were associated with behavioural, sensory, and autonomic indices of pain processing. Linear regression analyses examining associations between QSM/${R_{2}}^{*}$ measures and pain‐related outcomes are summarised in Table 2. Distinct region‐ and modality‐specific relationships were observed. Higher ${R_{2}}^{*}$ values of RN were associated with reduced CPM measures, including significant associations with absolute (*p* = 0.015) and relative (*p* = 0.022) CPM. In contrast, higher QSM values in the PAG were associated with greater temporal summation, as reflected by WUR (*p* = 0.009), and showed a trend‐like association with higher subjective pain sensitivity measured by PSQ‐minor (*p* = 0.072).

Autonomic reactivity during pain conditioning, assessed by PRD, was associated with ${R_{2}}^{*}$ in both (RN/PAG) regions. Higher ${R_{2}}^{*}$ values in the RN were associated with increased PRD during heat conditioning (*p* = 0.016), whereas higher ${R_{2}}^{*}$ values in the PAG were associated with increased PRD during both heat‐ and cold‐water conditioning (*p* = 0.023). No consistent associations were observed for GP measures.

## 4. Discussion

The focus of our prospective in vivo study was the investigation of whether quantitative MRI markers of regional brain iron, magnetic susceptibility, and ${R_{2}}^{*}$ values are associated with clinical pain‐modulatory processes in CP and M patients compared with HV. Across participants, regional iron‐sensitive MRI measures showed distinct meaningful associations with behavioural, sensory, and autonomic outcomes, linking iron content to multiple dimensions of pain processing. In contrast, regional iron measures showed limited ability to distinguish between clinical groups. These findings suggest that iron‐sensitive MRI captures inter‐individual differences in pain‐modulatory function across participants, in line with modern models of descending control and pain chronification [1, 2, 23, 24].

### 4.1. Lack of Group Differences in QSM and $R_{2}^{*}$‐Based Iron Levels

Across the GP, PAG, and RN, categorical group differences in iron content were small. Only QSM in the GP showed a group effect (*p* = 0.04), while PAG and RN did not differ between CP, M, and HV. This isolated GP group effect should be interpreted cautiously. Given the high iron content and pronounced variability of the GP across individuals, subtle group differences may emerge without clear functional relevance. Reports of deep grey matter iron changes have previously described variable findings without consistent associations with pain‐modulatory measures [25, 26], which is consistent with the absence of GP‐related behavioural or physiological correlations in the present study. More broadly, the absence of robust group differences aligns with the understanding that iron accumulation in deep brain structures is a gradual and heterogeneous process, shaped by age (CP 35.5 [27.8, 53.8]; M 34.0 [25.8, 48.3]; HV 32.5 [27.0, 45.0]), systemic and metabolic factors, and disease chronicity [3, 27–31]. Similar patterns of subtle and variable iron changes have been reported in migraine populations, with more pronounced effects typically observed in chronic migraine compared with episodic forms [7, 25, 26]. Because the M group here consisted almost entirely of episodic cases and the CP group exhibited heterogeneous aetiologies (11 patients with multi‐site/9 with single‐site pain localisation) and moderate symptom burden and duration of pain (CP 3.5 ± 0.89, EM 3.1 ± 1.65 years), pronounced group differences were not necessarily expected. Furthermore, both QSM and ${R_{2}}^{*}$ values show substantial natural inter‐individual variability even in healthy populations [29, 30, 32–35], which may depend on reconstruction pipelines and susceptibility offset handling in QSM [36]. Therefore, current findings suggest the view that iron‐sensitive MRI is more informative when examined dimensionally, in association with behavioural and physiological outcomes rather than purely categorical diagnoses. To our knowledge, no previous study has systematically examined both QSM and ${R_{2}}^{*}$ (1/${T_{2}}^{*}$) measures in patients with CP and migraine across multiple midbrain regions. A previous MRI study at 3T assessed QSM values in nine HV across five brain regions, including the RN and GP, and reported values that are consistent with those observed in our cohort [36].

### 4.2. RN: A Key Correlate of Descending Pain Inhibition

Although iron levels of RN did not differ between groups, ${R_{2}}^{*}$ results of RN showed the clearest and most consistent association with descending inhibition. Both absolute (*p* = 0.015) and relative CPM (*p* = 0.022) measures varied with ${R_{2}}^{*}$ results of RN, and post‐hoc group comparisons indicated differences between CP and HV (abs. *p* = 0.037, rel. *p* = 0.035) and CP and M (abs. *p* = 0.038, rel. *p* = 0.017). Behaviourally, migraine patients demonstrated the largest CPM effects, HV intermediate, and CP the smallest. This pattern is consistent with evidence that CP conditions are commonly associated with impaired CPM, whereas episodic migraine may show preserved or relatively intact descending inhibition outside acute attacks [37, 38]. Higher CPM values in this paradigm reflect stronger descending modulation of the test stimulus, consistent with the concept of CPM as an index of endogenous inhibitory capacity [9, 19–21, 23]. The association with ${R_{2}}^{*}$ results of RN indicates that RN’s microstructural properties correspond to inter‐individual differences in the efficiency of endogenous inhibitory control, rather than reflecting categorical differences between CP, migraine and HV groups. This is coherent with anatomical and functional work highlighting the RN as part of a cerebellar–brainstem network involved in motor control, sensorimotor integration, and nociceptive processing [39, 40]. Further studies implicate the RN in the modulation of neuropathic and inflammatory pain through cytokine and glutamatergic mechanisms [41–43]. Thus, RN, through the acquired ${R_{2}}^{*}$ measures, in our study cohort can be interpreted as a structural–metabolic correlate of descending inhibition, consistent with broader models of descending control in CP [23, 24].

### 4.3. PAG: A Central Hub for Pain Facilitation and Autonomic Modulation

The PAG did not show categorical iron differences; however, ${R_{2}}^{*}$ results in the PAG displayed key relationships with pain‐facilitatory and autonomic measures. QSM values of the PAG were associated with temporal summation (WUR, *p* = 0.009), and CP participants (−0.479 ± 0.92) exhibited greater summation than M (0.171 ± 1.11). Iron measures (${R_{2}}^{*}$) of the PAG were also related to PRD reactivity, including PRD during the cold‐water conditioning stimulus (*p* = 0.023). Additionally, a trend‐level association between QSM values of PAG and PSQ‐minor suggests that PAG microstructure may relate to subjective sensitivity to everyday pain (*p* = 0.072). These findings are consistent with the established role of the PAG as a key integrative hub in defensive, nociceptive, and autonomic control. Further studies show that the PAG coordinates defensive responses and integrates neuropeptidergic signalling relevant for pain and threat processing [4–6]. Neuroimaging work also demonstrates functionally distinct PAG subdivisions that differentially relate to threat, respiratory, and nociceptive processing [44, 45], and implicates PAG–brainstem networks in temporal summation of pain [40, 46]. Iron deposition in the PAG has been reported in chronic migraine and related headache conditions [7, 25, 26], highlighting its sensitivity to long‐standing nociceptive burden. In this context, our results suggest that PAG iron content is associated with inter‐individual differences in pain amplification and autonomic reactivity, rather than simply distinguishing diagnoses.

### 4.4. Complementary Sensitivities of QSM and $R_{2}^{*}$ Findings

QSM and ${R_{2}}^{*}$ findings yielded partially distinct patterns of association, reflecting their differing physical sensitivities. QSM technique primarily shows local magnetic susceptibility, which is strongly influenced by iron distribution, while ${R_{2}}^{*}$ additionally reflects microvascular and microstructural contributions, including myelin and tissue organisation [32–35]. Our current findings mirror this: PAG‐related associations (WUR, PSQ‐minor) were more pronounced for QSM, whereas CPM and certain mechanical QST relationships were stronger for ${R_{2}}^{*}$ in the RN. These complementary profiles highlight that multiple microstructural features contribute to individual differences in pain‐modulatory function, and that QSM and ${R_{2}}^{*}$ techniques work together, providing clearly distinct insights into the architecture of midbrain pain pathways [3, 32, 33].

### 4.5. Sensory Findings (QST): Peripheral and Central Sensitisation

QST revealed clear sensory differences between CP and M participants. CP showed lower HPT, consistent with heat hyperalgesia, and tended to exhibit greater MPS and increased temporal summation (WUR). These findings align with prior work demonstrating generalised hyperalgesia and altered sensory processing across CP conditions using DFNS‐based and related QST protocols [8, 22, 47, 48]. However, HPT did not correlate with iron content. With regard to underlying mechanisms of CP, heat hyperalgesia is thought to primarily reflect peripheral sensitisation [49–51], rather than reflecting midbrain microstructural variation. Only two QST measures, MPT and MPS, showed associations with RN iron measures (MPT: *p* = 0.02; MPS: *p* = 0.03), which is plausible given the RN’s role in sensorimotor and nociceptive integration [39, 40, 42, 43]. Overall, the dissociation between QST group differences and iron MRI associations supports the existence of distinct physiological domains: threshold‐based sensory processes vs central‐mediated modulatory processes captured by CPM, WUR, and PRD [8, 22–24, 47].

### 4.6. Autonomic Reactivity (PRD): Integrated PAG‐RN Contributions

PRD provides an objective index of autonomic and noradrenergic arousal during nociceptive stimulation and is closely linked to brainstem circuits involving the locus coeruleus (LC) and PAG. Unlike subjective pain ratings, PRD captures pain‐related autonomic reactivity independently of conscious report, supporting its validity as a complementary physiological marker of pain modulation. PRD responses did not differ significantly between groups, indicating considerable overlap in autonomic responses across the three cohorts. This is compatible with emerging literature using pupillometry as an objective marker of nociceptive processing, which also reports considerable variability even in HV [10]. In our study, however, important associations emerged between PRD and iron measures in PAG and RN. ${R_{2}}^{*}$ results of RN were associated with PRD during heat stimulation (*p* = 0.016), while ${R_{2}}^{*}$ results of PAG were linked to PRD during cold‐water conditioning (*p* = 0.023). These associations are consistent with the central autonomic control of nociception, in which both the PAG and RN contribute to sympathetic arousal and autonomic adjustments during painful or threatening sensory events. The PAG plays a central integrative role in defensive and autonomic regulation [2, 44, 45], including the modulation of pupil diameter through its interactions with brainstem nuclei involved in parasympathetic output, such as the Edinger–Westphal complex, and with arousal systems, including LC [2, 44]. Likewise, the RN is anatomically and functionally interconnected with cerebellar and brainstem structures that contribute to nociceptive processing and coordinated motor–autonomic responses [39, 40]. Together, these findings support the view that individual differences in autonomic reactivity, shown here by PRD, form part of a broader pain‐modulation profile that is better captured dimensionally than categorically.

### 4.7. Limitations

When interpreting these results, several limitations are important to mention. Although each group ultimately included 20 participants, the final sample size reflects the extensive screening and strict inclusion criteria that we applied. These requirements ensured that the three groups were highly comparable and free of confounding medical, psychiatric, or neurological conditions that could have influenced the results, but they also inevitably reduced the number of eligible participants. Another limitation relates to the precision required for ROI‐based analyses. Many ROIs were drawn across several deep brain structures, including very small nuclei such as the PAG. To ensure consistency, structured training was conducted beforehand, and ROI placement followed predefined anatomical landmarks established together with our experienced senior neuroradiologist (Sönke Peters). Even with these measures, some degree of variability is unavoidable when working at the limits of MRI. The cross‐sectional design of the study also limits conclusions about causality; we can describe associations, but we cannot determine whether iron differences influence pain modulation or vice versa. Iron regulation itself is shaped by a wide range of biological factors, including systemic physiological factors and genetic predispositions. Because these mechanisms are complex and not fully understood, some of the variability in QSM and ${R_{2}}^{*}$ values likely reflects natural inter‐individual differences that cannot be completely accounted for in a single study. In addition, behavioural and psychophysical measures naturally show some moment‐to‐moment variability, even when testing procedures are carefully standardised. Finally, although QSM and ${R_{2}}^{*}$ values are widely used as indirect markers of iron, their signals also reflect contributions from other tissue characteristics to a small degree. Modern reconstruction approaches, including background field correction, dipole inversion, and the use of anatomical priors, substantially enhance the iron sensitivity of these metrics. Nonetheless, a minor portion of the signal inevitably reflects other microstructural features. This is an inherent property of MRI‐based iron measures and should be considered when interpreting regional values.

## 5. Conclusion

Iron‐sensitive MRI measures captured meaningful inter‐individual variation across behavioural, sensory, and autonomic dimensions of pain processing. QSM and ${R_{2}}^{*}$‐specific associations involving the PAG and RN linked regional iron content to descending inhibition CPM, nociceptive gain (WUR), and autonomic reactivity (PRD), suggesting that microstructural variation within these nuclei relates to how individuals engage endogenous modulatory systems. ${R_{2}}^{*}$ RN was closely linked to CPM and MPT and MPS, while QSM PAG was associated with pain amplification (WUR); QSM PAG also revealed a trend‐level association with PSQ‐minor. Autonomic responses (PRD) were associated with ${R_{2}}^{*}$ in both regions (PAG/RN), reflecting their distinct roles within brainstem pain networks. Sensory findings such as reduced HPT in CP further highlight that central and peripheral sensitisation coexist alongside central modulatory differences. However, iron content showed limited ability to distinguish between clinical groups. Thus, iron‐sensitive MRI techniques appear more informative as a marker of individual pain‐modulatory physiology than as a categorical classifier of clinical groups. These results support the concept of an iron‐related physiological biotype of pain modulation, encouraging future longitudinal work to clarify its relevance for treatment response and pain chronification.

**Nomenclature**
ANOVA: Analysis of variance
AP: Anterior‐posterior
BL: Bright light
BW: Bandwidth
CAMERA: Cerebral abnormalities in migraine: an epidemiological risk analysis
CM: Chronic migraine
COVID: Corona virus disease
CP: Chronic pain
CPM: Conditioned pain modulation
CPT: Cold pain threshold
CS: Conditioning stimulus
DMA: Dynamic mechanical allodynia
DRIVE: Driven equilibrium
DSCF: Dwass–steel–critchlow–fligner
DFNS: Deutscher forschungsverbund neuropathischer schmerz e.V.
EM: Episodic migraine
FLAIR: Fluid attenuated inversion recovery
FOV: Field of view
GDPR: General data protection regulation
GP: Globus pallidus
GRE: Gradient‐echo
HPT: Heat pain threshold
HV: Healthy volunteers = healthy controls
ICHD‐3: International classification of headache disorders, 3rd edition
IQR: Interquartile range
KW: Kruskal–Wallis (test)
M: Migraine
MATLAB: Matrix laboratory based toolboxes
mFFE: Multi‐fast field echo
mN: Millinewton
ms: Milliseconds
MITK: Medical imaging toolkit kit (program)
MPT: Mechanical pain threshold
MPS: Mechanical pain sensitivity
MRI: Magnetic resonance imaging
NRS: Numerical rating scale
PACS: Picture archiving and communication system
PAG: Periaqueductal grey
PPM: Parts per million
PRD: Pupil reflex dilation
PSQ: Pain sensitivity questionnaire
PSQ‐minor: Pain sensitivity questionnaire minor subscale
PSQ‐moderate: Pain sensitivity questionnaire moderate subscale
PSQ‐total: Pain sensitivity questionnaire total score
QSM: Quantitative susceptibility mapping
QST: Quantitative sensory testing
RL: Right‐left
RN: Red nucleus
ROI: Region of interest
RVM: Rostroventromedial
${R_{2}}^{*}$
: ${\text{R}}_{2}^{*}$ mapping
SD: Standard deviation
TE: Time of echo
TFE: Turbo field echo
TI: Time of inversion
TR: Time of repetition
T_1_w: T_1_‐weighted imaging
T_2_w: T_2_‐weighted imaging
${T_{2}}^{*}$
: ${\text{T}}_{2}^{*}$ mapping
TS: Test stimulus
VISTA: Volume isotropic turbo spin‐echo acquisition
WFS: Water‐fat‐shift
WUR: Wind‐up ratio

## Author Contributions

Omar Ydlibi: writing–original draft, investigation, formal analysis, and data curation. Mona Salehi Ravesh: review and editing, supervision, formal analysis, resources, methodology, investigation, and conceptualisation. Florian Bernerth: review and editing and data curation. Burkhard Maedler: review and editing and resources. Olav Jansen: review and editing and resources. Janne Gierthmühlen: review and editing, supervision, resources, project administration, methodology, and conceptualisation. Sönke Peters: review and editing, supervision, resources, methodology, investigation, and conceptualisation.

## Funding

No funding was received for this manuscript.

Open access funding was enabled and organised by Projekt DEAL.

## Disclosure

All authors have agreed to the final submitted version.

## Ethics Statement

The study protocol was conducted in accordance with the Declaration of Helsinki (as revised in 2024) and approved by the local ethics committee of Christian‐Albrechts‐University of Kiel (No. D 441/21).

## Consent

Written informed consent for this prospective study was obtained from all patients to publish this paper.

## Conflicts of Interest

The authors declare no conflicts of interest.

## Data Availability

The data that support the findings of this study are available from the corresponding author upon reasonable request.
